# Efficacy and Safety of Lianhua Qingke Tablets in the Treatment of Mild and Common-Type COVID-19: A Randomized, Controlled, Multicenter Clinical Study

**DOI:** 10.1155/2022/8733598

**Published:** 2022-02-10

**Authors:** Ling Zhang, Lei Wu, Xiaolong Xu, Yadong Yuan, Rongmeng Jiang, Xixin Yan, Xin Zhang, Yong Gao, Huanxia Shang, Bo Lian, Jing Hu, Jianqiang Mei, Shucai Wu, Qingquan Liu

**Affiliations:** ^1^Hebei Chest Hospital, Shijiazhuang 050041, China; ^2^Hebei Hospital of Traditional Chinese Medicine, Shijiazhuang 050011, China; ^3^Beijing Hospital of Traditional Chinese Medicine, Capital Medical University, Beijing 100010, China; ^4^The Second Hospital of Hebei Medical University, Shijiazhuang 050000, China; ^5^Beijing Ditan Hospital, Capital Medical University, Beijing 100015, China

## Abstract

**Background:**

Lianhua Qingke (LH) tablets is an effective traditional Chinese medicine against various viral infections, especially in relieving coughing. However, its effects on COVID-19 are unknown.

**Methods:**

To examine the therapeutic effectiveness of LH tablets in COVID-19 patients with mild and common types, a randomized, multicenter, controlled study was carried out. COVID-19 cases were randomized to undergo routine treatment with or without LH tablets (4 tablets, three times a day) for 14 days. The primary endpoints were the rate of achieving clinical symptom resolution and the corresponding time.

**Results:**

There were 144 participants in the full analysis set (72 each in the LH and control groups). The LH group participants had elevated symptom alleviation rate at 14 days compared with control cases (FAS: 98.61% vs. 84.72%, *p* = 0.0026). In comparison with control group participants, the LH group participants had reduced median time to clinical symptom alleviation (median: 4 vs. 7 days, *p* < 0.0001). Higher resolution rates of coughing (98.44% vs. 84.51%, *p* = 0.0045) and expectoration (100% vs. 82.35%, *p* = 0.0268) were observed in the LH group. Times to recovery of fever (median: 2 vs. 3 days, *p* = 0.0007), coughing (median: 4 vs. 7 days, *p* < 0.0001), and expectoration (median: 3 vs. 6 days, *p* < 0.0001) were also notably shorter in the LH group. Moreover, the LH group had elevated improvement rates in chest computed tomography signs (FAS: 86.11% vs. 72.22%, *p* = 0.0402) and clinical cure at day 28 (FAS: 83.33% vs. 68.06%, *p* = 0.0326). However, no differences were found in the laboratory test and viral assay. Serious adverse events were not detected.

**Conclusion:**

These preliminary findings indicate LH tablets may be effective in symptomatic COVID-19, especially in relieving coughing. This trial was registered in Chinese Clinical Trial Registry (ChiCTR2100042069).

## 1. Introduction

In December 2019, coronavirus disease 2019 (COVID-19), resulting from infection by severe acute respiratory syndrome coronavirus 2 (SARS-CoV-2), emerged as a new pandemic that has continued to pose a severe threat to the global community [1]. As of April 2021, more than 146.8 million confirmed cases and 3.1 million deaths have been reported across 188 countries (World Health Organization, WHO). It has been reported that COVID-19 vaccines may have a limited effect in combating SARS-CoV-2 spread and protecting from symptomatic COVID-19 [2]. A recent solidarity trial by the WHO also reported that four most promising antiviral drugs, including remdesivir, hydroxychloroquine, lopinavir, and interferon regimens, are minimally effective in severe COVID-19 [3]. Therefore, there is still no specific remedy available to infected patients.

Coughing is considered a major clinical manifestation in COVID-19 patients. Pathological anatomy showed mucus and mucus-related thrombosis in the bronchial cavity, serous, fibrin exudate, and hyaline membrane formation in the alveolar cavity [4]. These viscous exudates block the airway, which seriously affects the ventilation function of the lung, reduces the efficacy of oxygen therapy and mechanical ventilation, and even causes asphyxial death [5]. In addition, coughing is a stigma for patients recovering from COVID-19, as a common post-COVID-19 syndrome symptom [6]. Based on reports and feedback from patients, many individuals are with post-COVID syndrome symptoms such as coughing dread reinfection, staying away from relatives and friends, and/or have trouble resuming work [7]. Therefore, taking effective measures and administering drugs to ensure airway patency and improve lung ventilation function has become an important way to improve the cure rate of COVID-19 cases. However, the current lack of effective anticoronavirus drugs is compounded by the scarcity of effective drugs for the treatment of the respiratory tract, especially targeting small airway mucus or sputum embolism [6]. Therefore, it is urgent to develop effective drugs to relieve clinical symptoms, especially cough and sputum.

LH tablets is a marketed patented Chinese medicine drug designed for treating respiratory diseases, especially those accompanied by cough and sputum. It was approved by the State Food and Drug Administration (SFDA No. 2010L00120) in 2010 and underwent a phase III clinical trial in 2014 with confirmed efficiency. LH tablets is mainly composed of thirteen herbs, including Mahuang (stem, Herba Ephedra), Sangbaipi (root bark, *Morus Alba* L.), Shigao (*Gypsum fibrosum*), Huangqin (root, *Scutellaria baicalensis*), Kuxingren (semen, armeniacae amarum), Lianqiao (fruit, *Forsythia suspensa*), Banxia (stem, *Pinellia ternate*), Zhebeimu (bulb, *Fritillaria thunbergii*), Qianhu (root, *Peucedanum praeruptorum*), Niubangzi (fruit, *Fructus arctii*), Jinyinhua (flos, *Lonicera japonica* Thunb.), Dahuang (root, Rhei Radix et Rhizoma), and Chenpi (pericarpium, *Citrus reticulata* Blanco.). The main function of LH is stopping cough. The indications of LH include acute tracheitis and bronchitis-caused cough and headache [8].

Considering COVID-19 symptoms and LH tablet indications, we hypothesized that LH might be beneficial for cases showing upper respiratory symptoms. Here, we conducted a randomized controlled study, aiming to examine the effectiveness of LH tablets in COVID-19.

## 2. Patients and Methods

### 2.1. Study Design and Patients

The current randomized, controlled, multicenter study examined COVID-19 cases diagnosed based on the Diagnosis and Treatment Program for Novel Coronavirus Infection Pneumonia (Trial Eighth Edition) released by the National Health Commission of China. This trial was planned and prepared since January 1, 2021, and started enrollment from January 14 to March 31, 2021. This study was performed in Thoracic Surgery Department, Tuberculosis Department, and Department of Neurology of the Hebei Chest Hospital and Second Hospital of Xingtai and Harbin Chest Hospital. During the outbreak of COVID-19 in Shijiazhuang, the whole Hebei Chest Hospital and Second Hospital of Xingtai were changed into COVID-19-designated hospitals, which only received and cured COVID-19 patients. This trial was approved by the Ethics Committee of Hebei Province Chest Hospital and was performed according to the GCP guidelines and the Declaration of Helsinki. Each patient or designated legal representative provided signed informed consent. This trial was registered in Chinese Clinical Trial Registry (ChiCTR2100042069).

Inclusion criteria were as follows: confirmed COVID-19 by virus testing; ≥18 years of age; fever, cough, expectoration, chest tightness, polypnea, and/or dyspnea at enrollment; and informed consent provided. Exclusion criteria were as follows: overt bacterial infection in the respiratory tract resulting from common pathologies, including primary immunodeficiency disease, acquired immunodeficiency syndrome, congenital respiratory malformation, congenital heart disease, gastroesophageal reflux disease, and abnormal lung development; asthma treated daily, chronic airway disease, respiratory bacterial infections (e.g., purulent tonsillitis), acute tracheobronchitis, sinusitis, otitis media, and further respiratory tract pathologies potentially affecting the trial's data analysis; common pulmonary diseases (e.g., severe pulmonary interstitial lesions and bronchiectasis) confirmed by chest CT; severe pneumonia requiring ventilator use; previous or present diseases potentially affecting trial participation or influencing study outcome, based on the investigator's judgment; pregnancy or lactation in women; participation in a clinical study in the past 3 months; and history of allergy to ≥2 drugs or foods or known allergy to the drug's constituents.

### 2.2. Clinical Classification

Patients were classified into mild, common, and severe cases. Mild: confirmed cases with fever (measured or subjective) or respiratory symptoms. Moderate: confirmed cases with fever (measured or subjective) or respiratory symptoms and radiographic evidence of pneumonia. Severe: confirmed cases with evidence including fever, respiratory symptoms, radiographic evidence of pneumonia, and at least one of the following symptoms: respiratory distress, RR ≥ 30 times/min; resting state, mean oxygen saturation ≤93%; and PaO_2_/FiO_2_ ≤ 300 mmHg (1 mmHg = 0.133 kPa).

### 2.3. CT Examination

The evaluation of CT chest film is to examine the lung CT imaging of patients by experienced radiologists. The improvement of chest CT imaging refers to the following: reduction of infiltration area, reduced abnormal area of radiology ground, alleviated glass opacity, and reduced nodule density.

### 2.4. Randomization and Grouping

A randomized, open-label, blank-controlled, multicenter trial was performed to estimate the efficacy and safety of LH tablets in mild and common-type COVID-19 cases. A total of 160 random numbers with block 4 were generated with SAS 9.4 (SAS Institute, USA). The computer-based 1 : 1 block randomization method was utilized to assign cases to the treatment and control groups, respectively. Consecutive patients were randomly recruited by attending clinicians until reaching the total amounts of cases assigned to that particular site. All attending clinicians have obtained the qualification of clinical trial from State Drug Administration.

### 2.5. Treatments

For routine management (control group), the totality of treatments followed the “Diagnosis and Treatment Program for Novel Coronavirus Infection Pneumonia (Trial Eighth Edition):” supportive oxygen therapy, administration of antivirals, and symptom management (Supplementary Table 1). In the LH treatment group, the patients received routine treatment and LH administration (4 tablets, thrice daily) for 14 days. LH tablets (Batch: A2005001) were provided and qualified by the Shijiazhuang Yiling Pharmaceutical Co., Ltd.

### 2.6. Endpoints

The primary endpoints were the rate of achieving clinical symptom resolution and the corresponding time within 14 treatment days. Clinical symptom resolution was reflected by the disappearance of major symptoms, including fever, cough, expectoration, chest tightness, polypnea, and dyspnea. Secondary endpoints included the rate of single symptom disappearance, time to major symptom disappearance, changes in color, sputum quality and amounts in cases showing expectoration, changes in oxygenation index (OI), aggravation rate within the treatment period (according to severe or critical illness definitions), rate of CT improvement, disease recovery rate, and time to and rate of the coronavirus test turning negative.

### 2.7. Safety

Adverse events (AEs), i.e., adverse medical events occurring during the whole trial, with or without causal association with the study drug, were recorded.

### 2.8. Statistical Analysis

Two-sided *p* < 0.05 indicated statistical significance, unless otherwise specified. Count data were presented as number or ratio; measurement data were presented as mean and standard deviation (SD) or range, and nonnormally distributed data were presented as median and 25th and 75th quartiles. Time-event data were described by median and 95% confidence interval (CI), with 25th and 75th quartiles. The *t*-test or Wilcoxon rank sum test was used for the comparison of quantitative data between groups. The chi-square test or exact probability test was employed for comparing categorical data. The Wilcoxon rank sum test or CMH test was used for grade data. The log-rank test was employed for time-event data.

To evaluate the main efficacy, the superiority test was used to compare the treatment and control groups, followed by the Newcombe–Wilson test. The log-rank test was performed to compare both groups for recovery time. Descriptive statistics of adverse events that occurred in this study were performed, and the numbers and percentages of patients with adverse events and adverse reactions in the treatment and control groups during the study period were provided. AEs' incidence rates between groups were compared by the *χ*^2^ test or Fisher exact probability test. SAS 9.4 (SAS Institute, USA) was utilized for statistical analysis.

## 3. Results

### 3.1. Characteristics of Patients

**Figure 1:**
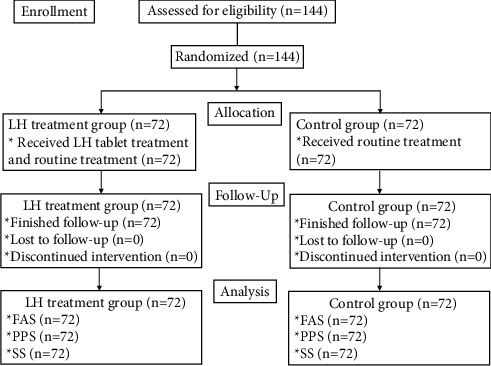
Study flowchart.

The flowchart of the trial is shown in Figure 1. A total of 144 COVID-19 cases were included and randomized in this study from January 12, 2021 to March 31, 2021, with 72 each in the control and LH treatment groups. In the end, 144 patients were included in this trial. Both groups were comparable in age, sex, temperature at onset (Table 1), and clinical characteristics (Table 2).

### 3.2. Primary Endpoints

**Figure 2:**
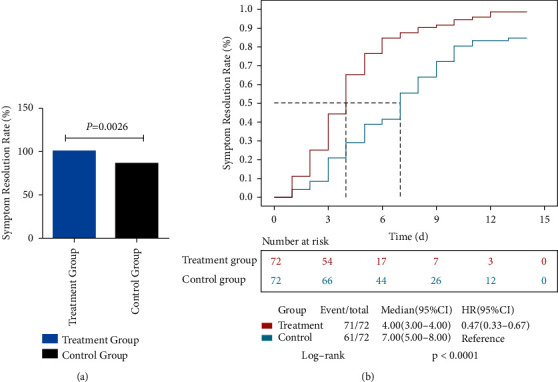
Rates of and times to symptom resolution.

The symptom recovery rate at 14 days was elevated in the LH treatment group compared with the control group (FAS: 98.61% vs. 84.72%, 95% CI: 4.98–24.00, *p* = 0.0026; PPS same as FAS) (Figure 2(a)). In addition, median time to symptom resolution was also detected in the LH treatment group in comparison with control cases (FAS: 4 days vs. 7 days, HR = 0.47, *p* < 0.0001) (Figure 2(b)).

### 3.3. Secondary Endpoints

**Figure 3:**
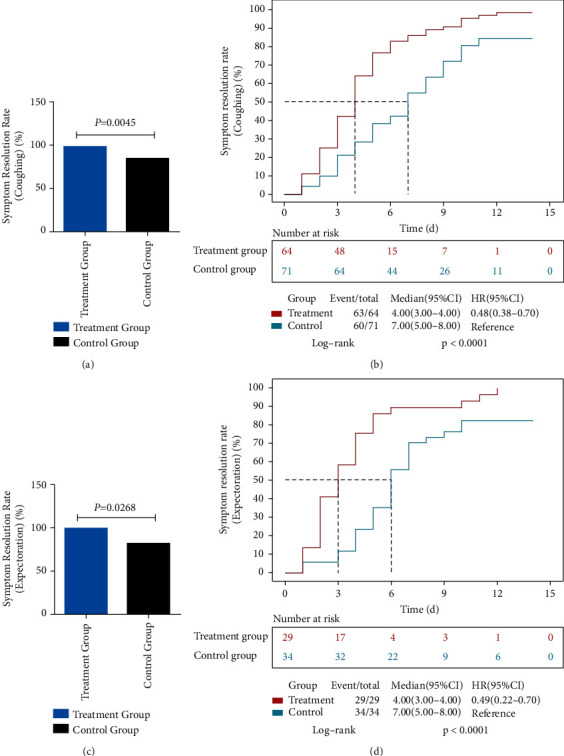
Rates of and times to respiratory symptom resolution.

Compared with the control group, the recovery rate of coughing at 14 days was higher in the treatment group (FAS: 98.44% vs. 84.51%, 95% CI: 4.46–24.17, *p* = 0.0045) (Figure 3(a)). The recovery rate of expectoration was also elevated in the treatment group (FAS: 100% vs. 82.35%, 95% CI: 2.71–33.52, *p* = 0.0268) (Figure 3(b)). By analyzing the time to achieving clinical symptom recovery, shorter times of cough (FAS: 4 days vs. 7 days, HR = 0.47, *p* < 0.0001) (Figure 3(c)) and expectoration (FAS: 4 days vs. 7 days, HR = 0.39, *p* < 0.0001) (Figure 3(d)) were observed in the treatment group compared with the control group.

**Figure 4:**
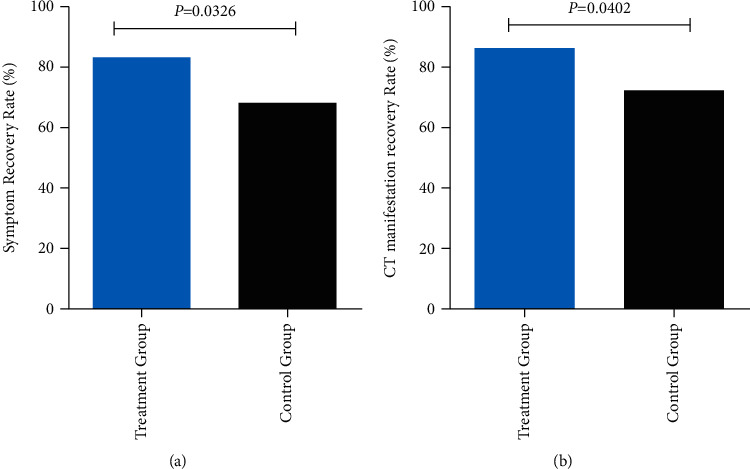
Rates of clinical cure and chest computed tomography manifestation improvement.

**Figure 5:**
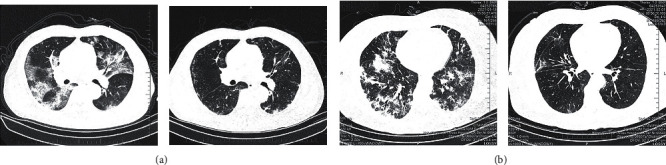
Computed tomography (CT) manifestations at enrolment and post-treatment.

The overall symptom resolution rate at 28 days was higher in the treatment group in comparison with the control group (FAS: 83.33% vs. 68.06%, HR = 0.94, *p* = 0.0326) (Figure 4(a)). The rate of recovery of chest CT signs also showed an increase in the treatment group (FAS: 86.11% vs. 72.22%, *p* = 0.0402) (Figure 4(b)). CT signs in two patients of the treatment group are shown in Figure 5. The CT results of patient 1 (Figure 5 (A1 and A2)) in the control group, to some extent, had a worse situation in lung tissue at day 12 compared with patient 2 in the treatment group (Figure 5 (B1 and B2)). More obvious consolidation of the left lower lobe and peripherals was observed in patient 1, while only slight consolidation of the right upper lobe in patient 2 was observed.

Taken together, LH tablets treatment resulted in an improved symptom resolution rate at 14 and 28 days, higher rate, and faster resolution of cough and expectoration and improved CT manifestations.

### 3.4. Safety

Safety findings are given in Table 3. AEs' incidence rates were similar in both groups, with 9.72% and 15.28% in the LH group and control group, respectively (*p* = 0.3135). No death was observed in either group. Of the AEs occurring in the treatment group, 6 of the 7 cases involved had no relationship with LH tablet administration.

## 4. Discussion

COVID-19 has seriously impacted the whole world since it was first discovered in Wuhan, China [9, 10]. Although in some countries the first wave of the pandemic has been under control, the second or third wave is occurring or expected to happen. Due to the limited supply of effective vaccines [11], methods to reduce disease spread such as avoiding crowds, wearing masks, and keeping physical distance are still important strategies to fight this infection [12, 13]. Similar to common cold and flu infections, coughing represents the main clinical sign of COVID-19 in its acute phase and continues in the postinfection phase [14]. Coughing not only brings pain but also promotes the spread of respiratory droplets in the community. Coughing patients may be stigmatized, leading to social isolation and widespread fear as a source of contagion, especially during the COVID-19 pandemic. Controlling COVID-19-related cough may reduce community transmission.

Unmet clinical needs for cough treatment have been broadly documented. Not a few patients with coughing express concerns of cough treatment [15, 16]. Up to now, treatment measures for acute and chronic cough in COVID-19 follow recently published guidelines [17, 18]. Although a few drugs for the relief of coughing are available on the market [19], there is still a lack of sound evidence demonstrating their therapeutic benefits for cough due to acute viral infections or pneumonia [20, 21]. In the routine treatment proposed by the Diagnosis and Treatment Program for Novel Coronavirus Infection Pneumonia (Trial Eighth Edition), no medicine but respiratory support methods are recommended. Corticosteroids, prescriptions that are used in acute lower respiratory tract infection and post-COVID patients, showed no improved outcome in shortening coughing time [22]. Anti-inflammatory drugs such as dexamethasone, which decreases mortality in COVID-19, showed no confirmed curative effect in relieving coughing [23].

LH, mainly composed of Maxing Shigan and Qingjin Huatan decoctions, removes heat from the respiratory system and helps reduce sputum production and viscosity, promoting discharge in viral or bacterial infections. The clinical observation showed promising therapeutic effects in COVID-19 patients, especially concerning cough relief. However, high-level evidence is lacking. Therefore, this RCT was carried out to estimate the effectiveness of LH tablets in COVID-19. According to above results, LH treatment significantly improved the clinical symptom resolution rate at 14 days. In comparison with control cases, LH treatment resulted in decreased median time to cough and expectoration resolution. Besides, higher rates of chest CT manifestation improvement were observed after LH administration. In two cases of control and LH treatment groups at enrollment, obvious bilateral pulmonary infiltrates and ground glass could be observed in CT results. After 12-day remedy, absorption of bilateral pulmonary infiltrates could be found in patients of both groups. However, consolidation of the left lower lobe and peripherals were observed in patients of the control group, while less consolidation of the right upper lobe was found in patients of the LH treatment group. However, LH treatment showed a limited synergistic effect in improving fever, chest tightness, and shortness of breath compared with the routine treatment. No differences were found in laboratory findings or viral burden. Seven cases in the treatment group and eleven cases in the control group were documented for adverse events. Gastrointestinal dysfunction and metabolic disorders were the most frequent adverse events according to organ classification. However, no severe events were documented and were definitely related to the treatment. No death event was reported.

Pharmacological research studies reported that the main ingredients of LH tablet may have antiviral and anti-inflammatory properties, contributing to the relief of respiratory symptoms and inflammatory responses. Dahuang and Gancao could suppress the membrane penetration of SARS-CoV and inhibit the replication of SARS-CoV [24]. Jinyinhua and Lianqiao possess the inhibitory effect in excessive inflammatory responses [25, 26]. Besides, it is documented that the most frequently used drug combination was Mahuang–Kuxingren, which contributes to the shorter cough time and better CT outcome of COVID-19 patients [27]. However, more mechanism research studies are needed to reveal how LH tablets is beneficial for COVID-19 patients.

There are some limitations in this study. The data to exhibit baseline clinical characteristics and adverse events were all enumeration data and only case number was recorded. No score methods were used for the evaluation of each symptom. Besides, 160 random numbers with block 4 were generated for the enrollment of patients in the beginning of this trial. Unexpectedly, the COVID-19 epidemic stopped before all 160 patients were included. However, 144 patients still met the need for our trial and followed the randomized principles. The baseline was also comparable. At last, we have to stress that all data from patients are recorded at day one of the enrollment, not the first time that disease occurred.

Overall, this RCT suggested LH tablets might be beneficial to symptomatic COVID-19 cases, especially in relieving cough and expectoration.

## Figures and Tables

**Table 1:** Baseline demographic data.

Variable	Treatment group *N* = 72	Control group *N* = 72	*P* value
Age (y, *x* ± *s*)	49.56 ± 14.88	52.81 ± 14.83	0.1915
Males (*N*, %)	23 (31.94%)	24 (33.33%)	0.8589
Temperature (°C, *x* ± s)	37.14 ± 0.81	37.01 ± 0.83	0.2164
Respiratory rate (bpm, x ± s)	19.75 ± 2.45	19.60 ± 2.15	0.6329
Pulse (times/min, *x* ± *s*)	79.72 ± 10.21	76.90 ± 9.02	0.3402
Systolic pressure (mmHg, *x* ± *s*)	124.75 ± 16.30	127.61 ± 13.14	0.0847
Diastolic pressure (mmHg, *x* ± *s*)	79.88 ± 9.51	80.53 ± 10.29	0.3990
Routine blood test			
Leukocyte count (*N*, *x* ± *s*)	5.09 ± 1.79	5.18 ± 1.50	0.5323
Neutrophil percentage (%, *x* ± *s*)	60.94 ± 12.46	61.22 ± 13.26	0.7064
Lymphocyte count (*N*, *x* ± *s*)	1.49 ± 0.60	1.49 ± 0.51	0.5987
Concomitant medications	56 (78.87%)	65 (90.28%)	0.0588

*P* value, comparison between the treatment group and the control group. *P* < 0.05 indicates a significant difference.

**Table 2:** Baseline clinical characteristics.

Symptom	Treatment group *N* = 72	Control group *N* = 72	*P* value
Fever (*N*, %)	34 (47.22%)	27 (37.5%)	0.3732
Fatigue (*N*, %)	20 (27.78%)	19 (26.39%)	0.8971
Cough (*N*, %)	64 (89.89%)	71 (98.61%)	0.4014
Expectoration (*N*, %)	29 (40.28%)	34 (47.22%)	0.7338
Short breath (*N*, %)	10 (13.89%)	8 (11.11%)	0.6232
Chest tightness (*N*, %)	11 (15.28%)	15 (20.83%)	0.3968
Dyspnea (*N*, %)	3 (4.17%)	3 (4.17%)	0.9908
Headache (*N*, %)	3 (4.17%)	3 (4.17%)	1.0000
Nausea (*N*, %)	5 (6.94%)	7 (9.72%)	0.5507
Vomit (*N*, %)	5 (6.94%)	5 (6.94%)	1.0000
Diarrhea (*N*, %)	4 (5.56%)	2 (2.78%)	0.4158
Anorexia (*N*, %)	6 (8.33%)	8 (11.11%)	0.5860
Dry rale (*N*, %)	0 (0%)	1 (1.39%)	0.3241
Wet rale (*N*, %)	1 (1.39%)	1 (1.39%)	1.0000

*P* value, comparison between the treatment group and the control group. *P* < 0.05 indicates a significant difference.

**Table 3:** Adverse events.

Adverse event	Treatment group *N* = 72	Control group *N* = 72	*P* value
Total (*N*, %)	7 (9.72%)	11 (15.28%)	0.3135
Heart dysfunction (*N*, %)	0 (0%)	1 (1.39%)	
Gastrointestinal dysfunction (*N*, %)	1 (1.39%)	6 (8.33%)	
Hepatobiliary dysfunction (*N*, %)	1 (1.39%)	1 (1.39%)	
Infections (*N*, %)	1 (1.39%)	1 (1.39%)	
Metabolic disorders (*N*, %)	3 (4.17%)	2 (2.78%)	
Neurological disorders (*N*, %)	1 (1.39%)	0 (0%)	
Severity^[1]^			0.3889
Mild (*N*, %)	6 (8.33)	11 (15.28)	
Moderate (*N*, %)	1 (1.39)	0 (0.00)	
Severe (*N*, %)	0 (0.00)	0 (0.00)	
Related to treatment			0.7484
Definitely related	0 (0.00)	0 (0.00)	
Probably related	0 (0.00)	0 (0.00)	
Definitely not related	1 (1.39)	1 (1.39)	
Probably not related	6 (8.33)	8 (11.11)	
Not sure	0 (0.00)	2 (2.78)	
Death (*N*, %)	0 (0.00)	0 (0.00)	1.0000

*P* value, comparison between the treatment group and the control group. *P* < 0.05 indicates a significant difference. ^[1]^Definition of severity: mild, confirmed cases with fever (measured or subjective) or respiratory symptoms; moderate, confirmed cases with fever (measured or subjective) or respiratory symptoms and radiographic evidence of pneumonia; severe, confirmed cases with evidence including fever, respiratory symptoms, radiographic evidence of pneumonia, and at least one of the following symptoms: respiratory distress, RR ≥30 times/min; resting state, mean oxygen saturation ≤93%; and PaO_2_/FiO_2_ ≤ 300 mmHg (1 mmHg = 0.133 kPa).

## Data Availability

The datasets used and/or analyzed in this study are available from the corresponding author upon request.

## Abbreviations

COVID-19: Coronavirus disease 2019
LH: Lianhua Qingke
SARS-CoV-2: Severe acute respiratory syndrome coronavirus 2
WHO: World Health Organization
AEs: Adverse events
CT: Computed tomography
FAS: Full analysis set
PPS: Per protocol set
SS: Safety set.

## Supplementary Materials

Supplementary Materials Table S1. Routine treatment in each group. CONSORT 2010 checklist of information to include when reporting a randomised trial.
