# Supplementary material for: Efficacy and Safety of Lianhua Qingke Tablets in the Treatment of Mild and Common-Type COVID-19: A Randomized, Controlled, Multicenter Clinical Study
Source: Evid Based Complement Alternat Med. 2022 Feb 10;2022:8733598. doi: 10.1155/2022/8733598 (PMC8853799; doi:10.1155/2022/8733598)
Supplement: Supplementary Materials — Table S1. Routine treatment in each group. CONSORT 2010 checklist of information to include when reporting a randomised trial. [file 8733598.f1.zip › 8733598.f1/CONSORT Checklist (1).pdf]

CONSORT 2010 checklist of information to include when reporting a randomised trial*

Section/Topic	Item No	Checklist item	Reported on Page Number/Line Number	Reported on Section/Paragraph
**Title and abstract**				
	1a	Identification as a randomised trial in the title	Page1, Line 1	Title Page
	1b	Structured summary of trial design, methods, results, and conclusions (for specific guidance see Table 2)	Page2, Line 25	Abstract
**Introduction**				
Background and objectives	2a	Scientific background and explanation of rationale	Page3, Line55	Introduction
	2b	Specific objectives or hypotheses	Page5, Line 96	Introduction
**Methods**				
Trial design	3a	Description of trial design (such as parallel, factorial) including allocation ratio	Page5, Line101	Methods/Study design and
	3b	Important changes to methods after trial commencement (such as eligibility criteria), with reasons	Not applicable	Not applicable
Participants	4a	Eligibility criteria for participants	Page6, Line113	Methods/Study design and
	4b	Settings and locations where the data were collected	Page5, Line105	Methods/Study design and
Interventions	5	The interventions for each group with sufficient details to allow replication, including how and when they were actually administered	Page7, Line151	Methods/Treatments
Outcomes	6a	Completely defined pre-specified primary and secondary outcome measures, including how and when they were assessed	Page7, Line158	Methods/Endpoints
	6b	Any changes to trial outcomes after the trial commenced, with reasons	Not applicable	Not applicable
Sample size	7a	How sample size was determined	Page7, Line142	Methods/Randomization
	7b	When applicable, explanation of any interim analyses and stopping guidelines	Page6, Line113	Methods/Study design and
Randomisation:				
Sequence generation	8a	Method used to generate the random allocation sequence	Page7, Line142	Methods/Randomization
	8b	Type of randomisation; details of any restriction (such as blocking and block size)	Page7, Line142	Methods/Randomization
Allocation concealment mechanism	9	Mechanism used to implement the random allocation sequence (such as sequentially numbered containers), describing any steps taken to conceal the sequence until interventions were assigned	Page7, Line142	Methods/Randomization and grouping

Implementation	10	Who generated the random allocation sequence, who enrolled participants, and who assigned participants to interventions	Page7, Line142	Methods/Randomization and grouping
Blinding	11a	If done, who was blinded after assignment to interventions (for example, participants, care providers, those assessing outcomes) and how	No blinding	No blinding
	11b	If relevant, description of the similarity of interventions	No blinding	No blinding
Statistical methods	12a	Statistical methods used to compare groups for primary and secondary outcomes	Page8, Line171	Methods/Statistical [illegible]
	12b	Methods for additional analyses, such as subgroup analyses and adjusted analyses	Page8, Line183	Methods/Statistical [illegible]
**Results**				
Participant flow (a diagram is strongly recommended)	13a	For each group, the numbers of participants who were randomly assigned, received intended treatment, and were analysed for the primary outcome	Page9, Line190	Results/Characteristics of the patients
	13b	For each group, losses and exclusions after randomisation, together with reasons	Page9, Line193	Results/Characteristics of [illegible]
Recruitment	14a	Dates defining the periods of recruitment and follow-up	Page9, Line192	Results/Characteristics of [illegible]
	14b	Why the trial ended or was stopped	Not applicable	Not applicable
Baseline data	15	A table showing baseline demographic and clinical characteristics for each group	Page9, Line195	Results/Characteristics of [illegible]
Numbers analysed	16	For each group, number of participants (denominator) included in each analysis and whether the analysis was by original assigned groups	Page9, Line191	Results/Characteristics of the patients
Outcomes and estimation	17a	For each primary and secondary outcome, results for each group, and the estimated effect size and its precision (such as 95% confidence interval)	Page9, Line196	Results/Primary endpoints
	17b	For binary outcomes, presentation of both absolute and relative effect sizes is recommended	Page9, Line196	Results/Primary endpoints
Ancillary analyses	18	Results of any other analyses performed, including subgroup analyses and adjusted analyses, distinguishing pre-specified from exploratory	Page9, Line202	Results/Secondary endpoints
Harms	19	All important harms or unintended effects in each group (for specific guidance see CONSORT for harms)	Page10, Line219	Results/Safety
**Discussion**				
Limitations	20	Trial limitations, addressing sources of potential bias, imprecision, and, if relevant, multiplicity of analyses	Not applicable	Not applicable
Generalisability	21	Generalisability (external validity, applicability) of the trial findings	Page11, Line226	Discussion
Interpretation	22	Interpretation consistent with results, balancing benefits and harms, and considering other relevant evidence	Page12, Line251	Discussion
**Other information**				
Registration	23	Registration number and name of trial registry	Page5, Line111	Methods/Study design and [illegible]

Protocol	24	Where the full trial protocol can be accessed, if available	http://www.chictr.org.cn/sh	http://www.chictr.org.cn/sh
Funding	25	Sources of funding and other support (such as supply of drugs), role of funders	Page14, Line304	FUNDING

*We strongly recommend reading this statement in conjunction with the CONSORT 2010 Explanation and Elaboration for important clarifications on all the items. If relevant, we also recommend reading CONSORT extensions for cluster randomised trials, non-inferiority and equivalence trials, non-pharmacological treatments, herbal interventions, and pragmatic trials. Additional extensions are forthcoming: for those and for up to date references relevant to this checklist, see www.consort-statement.org.

Table 2 Items to include when reporting a randomized trial in a journal or conference abstract

Item	Description	Reported on Page Number/Line Number	Reported on Section/Paragraph
Title	Identification of the study as randomized	Page1, Line1	Title Page
Authors *	Contact details for the corresponding author	Page1, Line4	Title Page
Trial design	Description of the trial design (e.g. parallel, cluster, non-inferiority)	Page2, Line26	Abstract
**Methods**			
Participants	Eligibility criteria for participants and the settings where the data were collected	Page6, Line113	Methods
Interventions	Interventions intended for each group	Page7, Line151	Methods/Treatments
Objective	Specific objective or hypothesis	Page5, Line 96	Introduction
Outcome	Clearly defined primary outcome for this report	Page9, Line196	Results/Primary endpoints
Randomization	How participants were allocated to interventions	Page7, Line142	Methods/Randomization
Blinding (masking)	Whether or not participants, care givers, and those assessing the outcomes were blinded to group assignment	No blinding	No blinding
**Results**			
Numbers randomized	Number of participants randomized to each group	Page9, Line190	Results/Characteristics of
Recruitment	Trial status	Page9, Line192	Results/Characteristics of
Numbers analysed	Number of participants analysed in each group	Page9, Line190	Results/Characteristics of
Outcome	For the primary outcome, a result for each group and the estimated effect size and its precision	Page9, Line196	Results/Primary endpoints
Harms	Important adverse events or side effects	Page10, Line219	Results/Safety

Conclusions	General interpretation of the results	Page11, Line226	Discussion
Trial registration	Registration number and name of trial register	Page5, Line111	Results/Study design and [illegible]
Funding	Source of funding	Page14, Line304	Funding

** this item is specific to conference abstracts*

From: Hopewell S, Clarke M, Moher D, et al. CONSORT for reporting randomized controlled trials in journal and conference abstracts: explanation and elaboration. PLoS Med. 2008;5(1):e20

Please leave this space alone as it will be supplemented by the editorial office when needed.
