# Supplementary material for: Efficacy and Safety of Lianhua Qingke Tablets in the Treatment of Mild and Common-Type COVID-19: A Randomized, Controlled, Multicenter Clinical Study
Source: Evid Based Complement Alternat Med. 2022 Feb 10;2022:8733598. doi: 10.1155/2022/8733598 (PMC8853799; doi:10.1155/2022/8733598)
Supplement: Supplementary Materials — Table S1. Routine treatment in each group. CONSORT 2010 checklist of information to include when reporting a randomised trial. [file 8733598.f1.zip › 8733598.f1/Supplementary material.doc]

**Table S1 Routine treatment in each group**

| **Variables** | **LH treatment group**  **cases** | **events** | **Control group**  **cases** | **events** |
| --- | --- | --- | --- | --- |
| **For respiratory system** |  |  |  |  |
| **Cough and cold drugs**  (Dextromethorphan Hydrobromide; Pseudoephedrine hydrochloride; chlorpheniramine; Ambroxol hydrochloride; Acetylcysteine; Carboxymethylstein) | 6 | 9 | 2 | 3 |
| **Drugs for obstructive tracheal disease**  (Formoterol fumarate; Budesonide; Montelukast sodium; Ipratropium bromide) | 3 | 3 | 0 | 0 |
| **Others**  (Difficult to classify) | 27 | 32 | 29 | 33 |
| **Systemic anti-infective Drugs** |  |  |  |  |
| **Antiviral drugs**  (Entecavir; Arbidol hydrochloride) | 11 | 11 | 9 | 10 |
| **Antibiotics**  (Moxifloxacin; Moxifloxacin hydrochloride) | 0 | 0 | 2 | 2 |
| **For nervous system** |  |  |  |  |
| **Analgesics**  (Acetaminophen; Dextromethorphan hydrobromide; Pseudoephedrine hydrochloride; Chlorpheniramine; Aspisol; Aspirin) | 11 | 14 | 3 | 5 |
| **Antipsychotics**  (Aripiprazole; Eszopiclone) | 2 | 2 | 0 | 0 |
| **Others**  (Betahistine mesylate; Mecobalamin) | 1 | 1 | 0 | 0 |
| **Antitumor and immunomodulatory drugs** |  |  |  |  |
| **Immune enhancer**  (Thymus faxin; Recombinant interferon α2b) | 6 | 7 | 3 | 3 |
| **For blood and hematopoietic organs** |  |  |  |  |
| **Blood substitutes and perfusates**  (Sodium chloride; Sodium bicarbonate) | 3 | 3 | 0 | 0 |
| **Antithrombotic drugs**  (Clopidogrel bisulfate; Nadroparin calcium) | 1 | 1 | 0 | 0 |
| **For musculoskeletal system** |  |  |  |  |
| **Anti-inflammatory and anti-rheumatic drugs**  (Ibuprofen; Aceclofenac; Etoricoxib; Naproxen sodium) | 3 | 3 | 0 | 0 |
| **Digestive and metabolic system drugs** |  |  |  |  |
| **Mineral supplements**  (Potassium chloride) | 2 | 2 | 0 | 0 |
